# Supplementary material for: Cardiolipin inhibits the non-canonical inflammasome by preventing LPS binding to caspase-4/11
Source: EMBO J. 2025 Jul 16;44(16):4419–42. doi: 10.1038/s44318-025-00507-z (PMC12361528; doi:10.1038/s44318-025-00507-z)
Supplement: Supplementary file 1 — Appendix [file 44318_2025_507_MOESM1_ESM.pdf]

Appendix for

Cardiolipin inhibits the non-canonical inflammasome by preventing LPS binding to caspase-4/11

Table of Contents

Appendix Figure S1: RS-LPS induces TNF secretion in BMDM but not in HMDM, and inhibits LPS-induced TNF secretion in both HMDM and BMDM 2

Appendix Figure S2: CL inhibits the non-canonical inflammasome with an IC50 of 5 uM 3

Appendix Figure S3: CL inhibits the noncanonical inflammasome regardless of the LPS origin 4

Appendix Figure S4: CL does not affect basal full-length CASP4 expression while restoring iLPS-induced decrease - Western Blot Quantification related to Figure 2 ... 5

Appendix Figure S5: CL decreases iLPS-induced cleaved but not full-length CASP11- Western Blot Quantification related to Figure 2 6

Appendix Figure S6: CL is internalised by murine macrophages. 7

Appendix Figure S7: CL is internalised and colocalises with mitochondria in human macrophages. 8

Appendix Figure S8: CL inhibits the non-canonical inflammasome independently of GBPs. 9

Appendix Figure S9: CL does not inhibit TNF secretion induced by 1 ug/mL PA-LPS, but does suppress CASP11-dependent IL-1β secretion. 10

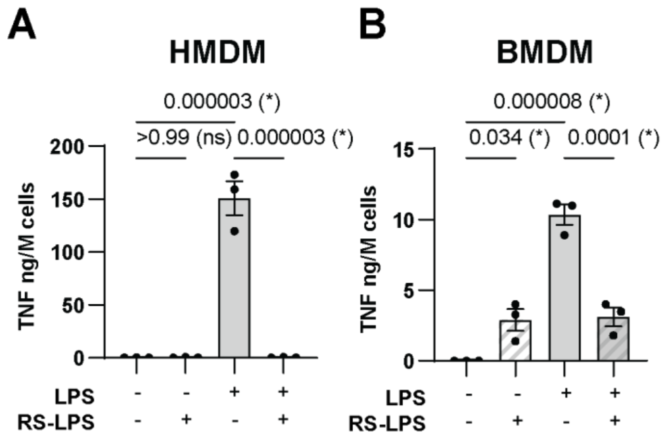

Appendix Figure S1: RS-LPS induces TNF secretion in BMDM but not in HMDM, and inhibits LPS-induced TNF secretion in both HMDM and BMDM.

HMDM (A) or BMDM (B) were incubated for 4 hours with 0.1 µg/mL LPS in the absence or presence of 2 µg/mL RS-LPS. TNF was quantified in cell supernatants by ELISA.

Data information: Each symbol is the mean of three technical replicates from an independent biological replicate. Bars are the mean of three independent biological replicates (n = 3) ± SEM.

Statistical analysis: Data were verified for normality using a Shapiro-Wilk test and analysed by one-way ANOVA Šídák's multiple comparisons test. P-values are reported in the figure. Significant difference for $p<0.05$ (*), not significant $p\geq0.05$ (ns).

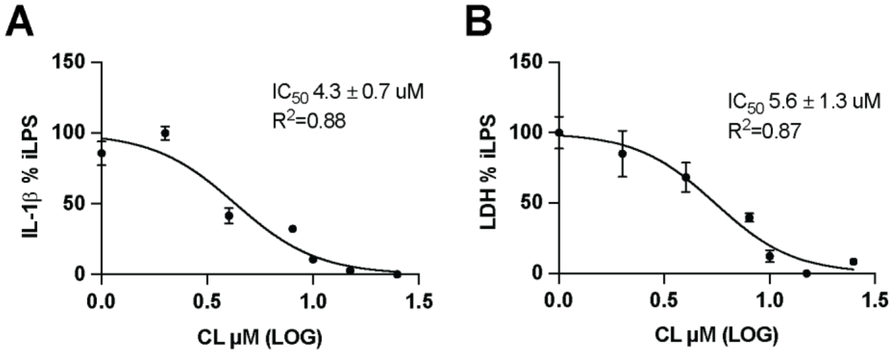

Appendix Figure S2: CL inhibits the non-canonical inflammasome with an IC50 of 5 uM.

BMDM were incubated for 4 hours with 1 μg/mL Pam3CSK4. Cell culture medium was then replaced with OptiMEM plus 0.5% FuGENE HD complexed with LPS derived from EC-B4, in the presence of increasing CL concentrations (1 to 30 μM). Cells were incubated for 18 hours. Cleaved IL-1β was quantified in cell supernatants by ELISA (A). LDH release was quantified by cytotoxicity assay (B).

Data information: Each symbol is the mean of three biological replicate (n = 3) ± SEM.

Statistical analysis: Dose–response curves were fitted using a four-parameter logistic (4PL) nonlinear regression model with variable slope in GraphPad Prism. IC_{50} values were derived from the fitted curves and are reported as mean ± SEM. The goodness of fit was assessed by R^2 values.

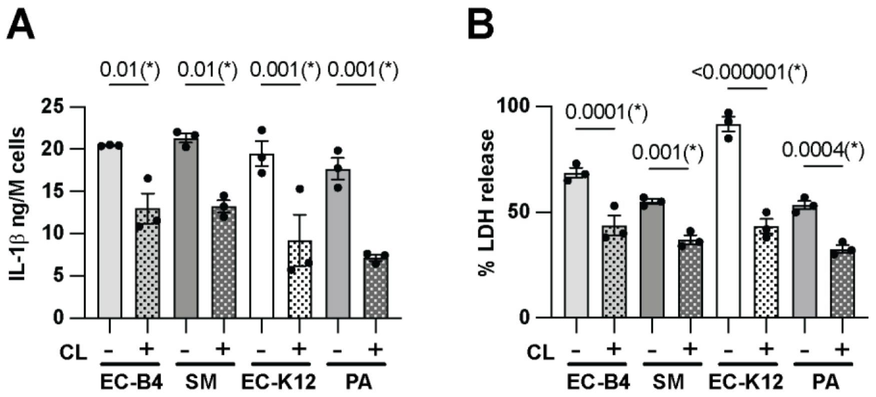

Appendix Figure S3: CL inhibits the noncanonical inflammasome regardless of the LPS origin.

BMDM were incubated for 4 hours with 1 µg/mL Pam_3CSK4. Cell culture medium was then replaced with OptiMEM plus 0.5% FuGENE HD complexed with LPS derived from *Escherichia coli* B4 strain (EC-B4), *Escherichia coli* K12 strain (EC-K12), *Salmonella minnesota* R595 (SM), or *Pseudomonas aeruginosa* (PA) in the presence of HEPES (-) or 10 µM 18:2 CL. Cells were incubated for 18 hours. Cleaved IL-1β was quantified in cell supernatants by ELISA (A). LDH release was quantified by cytotoxicity assay (B).

Data information: Each symbol is the mean of three technical replicates from an independent biological replicate. Bars are the mean of three independent biological replicates (n = 3) ± SEM.

Statistical analysis: Data were verified for normality using a Shapiro-Wilk test and analysed by one-way ANOVA. P-values are reported above the bars. Significant difference for $p<0.05$ (*), not significant $p\geq0.05$ (ns).

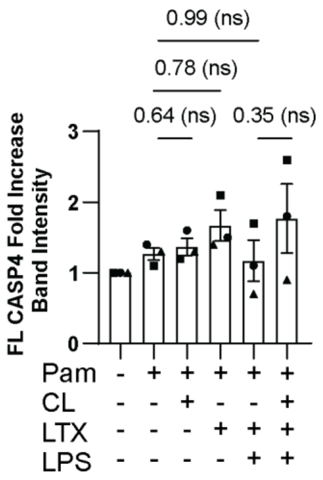

Appendix Figure S4: CL does not affect basal full-length CASP4 expression while restoring iLPS-induced decrease - Western Blot Quantification related to Figure 2.

HMDM were incubated for 4 hours with or without 1 µg/mL Pam_3CSK_4. Cell culture medium was then replaced with OptiMEM, LTX 0.25% v/v, 10 µM CL, or 2 µg/mL of LPS complexed with 0.25% v/v LTX in the presence of HEPES vehicle or 10 µM CL. Cells were incubated for 4 hours. The amount of full-length CASP4 was assessed in mixed supernatants and lysates by western blot. Band intensity was quantified using ImageLab (BioRad) (adjusted volumes) and reported here as fold increase compared to untreated cells.

Data information: Each symbol is the measure of an independent biological replicate. Bars are the mean of three independent biological replicates (n = 3) ± SEM.

Statistical analysis: Data were verified for normality using a Shapiro-Wilk test and analysed by repeated measures (RM) one-way ANOVA Šídák's multiple comparisons test (paired). P-values are reported in the figure. Significant difference for $p<0.05$ (*), not significant $p\geq0.05$ (ns).

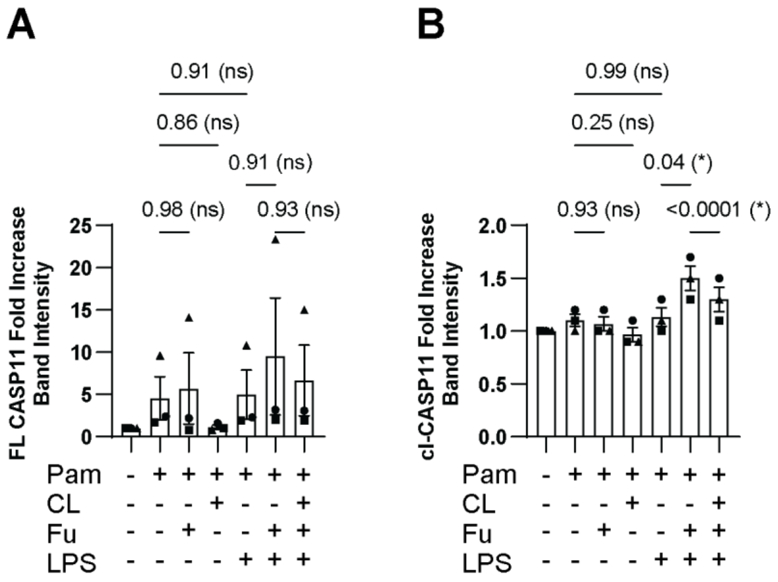

Appendix Figure S5: CL decreases iLPS-induced cleaved (cl) but not full-length CASP11- Western Blot Quantification related to Figure 2.

BMDM were incubated for 4 hours with or without 1 µg/mL Pam_3CSK_4. Cell culture medium was then replaced with OptiMEM, FuGENE HD 0.5% v/v, 10 µM CL, or 2 µg/mL of LPS alone or complexed with 0.5% FuGENE HD in the presence of HEPES vehicle or 10 µM CL. Cells were incubated for 4 hours. The amount of full-length (FL) (A) and cleaved (cl) (B) CASP11 was assessed in mixed supernatants and lysates by western blot. Band intensity was quantified using ImageLab (BioRad) (adjusted volumes) and reported here as fold increase compared to untreated cells.

Data information: Each symbol is the measure of an independent biological replicate. Bars are the mean of three independent biological replicates (n = 3) ± SEM.

Statistical analysis: Data were verified for normality using a Shapiro-Wilk test and analysed by repeated measures (RM) one-way ANOVA Šídák's multiple comparisons test (paired). P-values are reported in the figure. Significant difference for $p<0.05$ (*), not significant $p\geq0.05$ (ns).

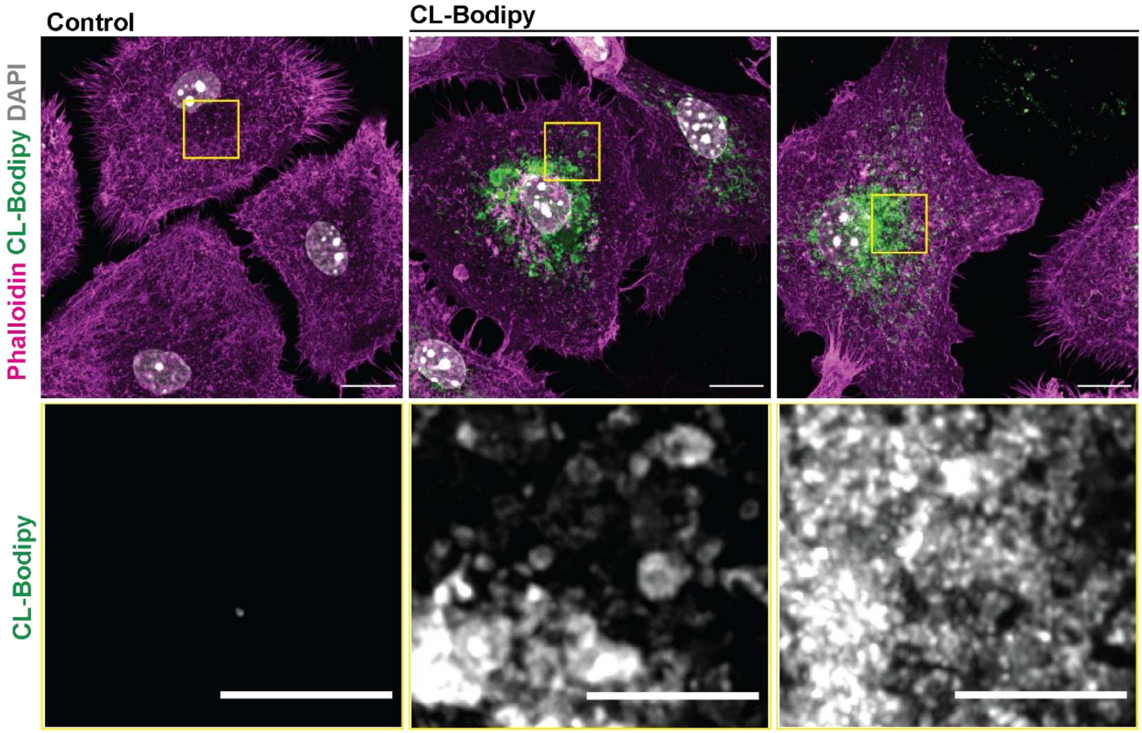

Appendix Figure S6: CL is internalised by murine macrophages.

Fixed-Airyscan confocal imaging of wild-type BMDM primed for 4 hours with 1 µg/mL Pam_3CSK_4, and incubated with HEPES (Control) or 10 µM of TopFluor CL (CL-BODIPY) for a further 18 hours. Macrophages were stained with the actin probe phalloidin (magenta), CL-BODIPY (green) and DAPI (grey).

Data information: Images are maximum intensity projections of Z-stack acquisitions. Scale bar = 10 µm. Scale bar inset = 5 µm. Images are representative of three independent experiments (n = 3).

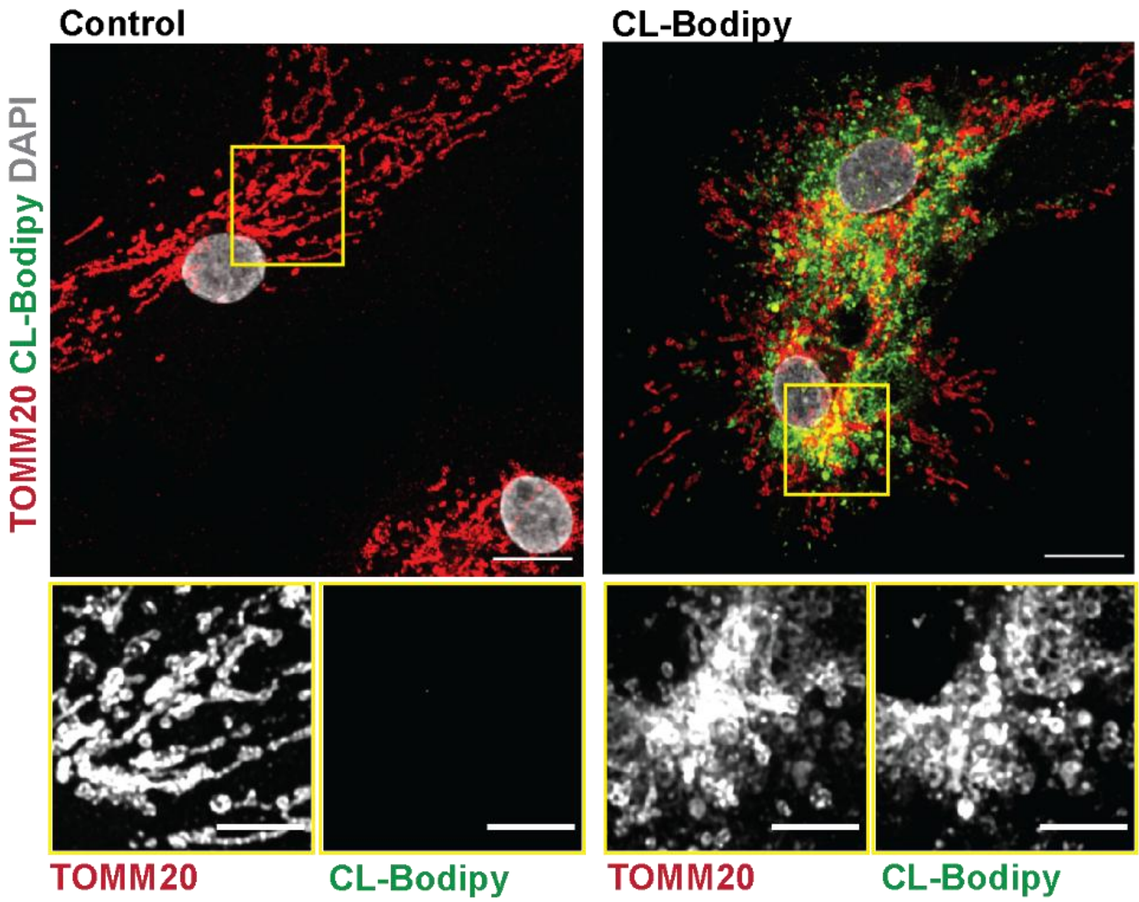

Appendix Figure S7: CL is internalised and colocalises with mitochondria in human macrophages.

Fixed-Airyscan confocal imaging of HMDM primed for 4 hours with 1 µg/mL Pam_3CSK_4 and incubated with HEPES (Control) or 10 µM of TopFluor CL (CL-BODIPY) for a further 18 hours. Macrophages were immunostained with TOMM20 (red), CL-BODIPY (green), and DAPI (grey).

Data information: Images are maximum intensity projections of Z-stack acquisitions. Scale bar = 10 µm. Scale bar inset = 5 µm. Images are representative of three independent experiments (n = 3).

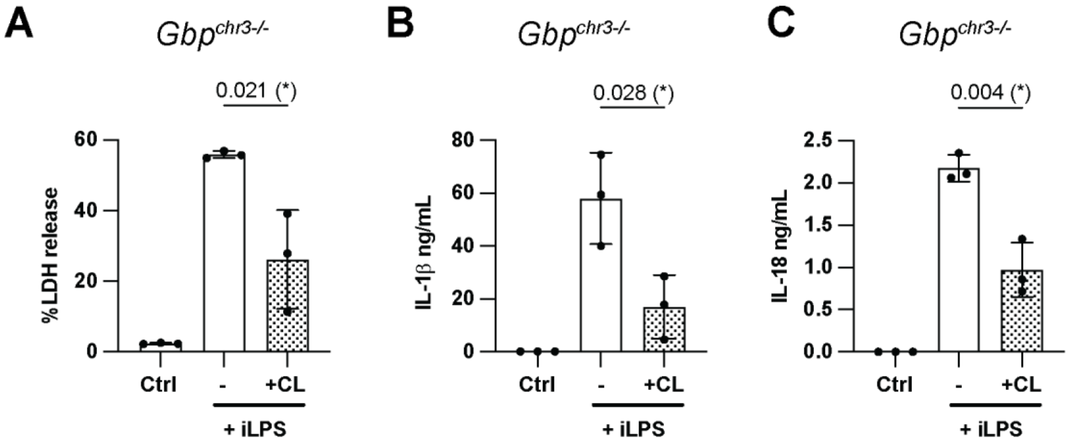

Appendix Figure S8: CL inhibits the non-canonical inflammasome independently of GBPs.

BMDM from *Gbp*$^{ch3-/-}$ mice were incubated for 4 hours with 1 µg/mL Pam_3CSK_4. Cell culture medium was then replaced with OptiMEM (ctrl) or LPS complexed with 1.2% Xfect in the presence of HEPES (-) or 10 µM CL. Cells were incubated for 4 hours. LDH release was quantified by cytotoxicity assay (A). Cleaved IL-1β (B) and total IL-18 (C) were quantified in cell supernatants by ELISA.

Statistical analysis: Data were verified for normality using a Shapiro-Wilk test and analysed by unpaired t-test. Adjusted p-values are reported above bars. Statistical significance was defined as follows: significant difference for p<0.05 (*), not significant for p≥0.05 (ns).

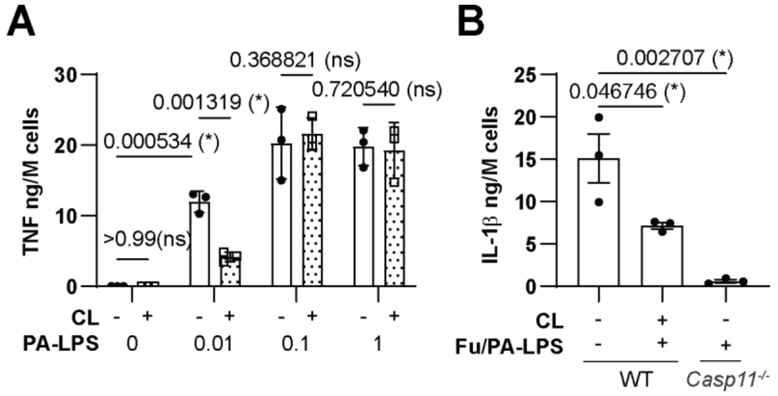

Appendix Figure S9: CL does not inhibit TNF secretion induced by 1 µg/mL PA-LPS, but does suppress CASP11-dependent IL-1β secretion.

A BMDM were incubated for 4 hours with increasing concentration of PA-LPS in the absence or presence of 10 µM CL. TNF was quantified in cell supernatants by ELISA.

B WT or *Casp11*$^{-/-}$ BMDM were incubated for 4 hours with 1 µg/mL Pam3CSK4. Cell culture medium was then replaced with OptiMEM plus 0.5% FuGENE complexed with 1 µg/mL PA- LPS (Fu/PA-LPS), in the absence or presence of 10 µM CL . Cells were incubated for 18 hours. Cleaved IL-1β was quantified in cell supernatants by ELISA.

Data information: Each symbol is the mean of three technical replicates from an independent biological replicate. Bars are the mean of three or more independent biological replicates (n = 3) ± SEM.

Statistical analysis: Data were verified for normality using a Shapiro-Wilk test and analysed by **A** two-way ANOVA Tukey's multiple comparisons test, and **B** one-way ANOVA Šídák's multiple comparisons test. P-values are reported in the figure. Significant difference for p<0.05 (*), not significant p≥0.05 (ns).
