# Supplementary figures and images for: Cardiolipin inhibits the non-canonical inflammasome by preventing LPS binding to caspase-4/11
Source: EMBO J. 2025 Jul 16;44(16):4419–42. doi: 10.1038/s44318-025-00507-z (PMC12361528; doi:10.1038/s44318-025-00507-z)

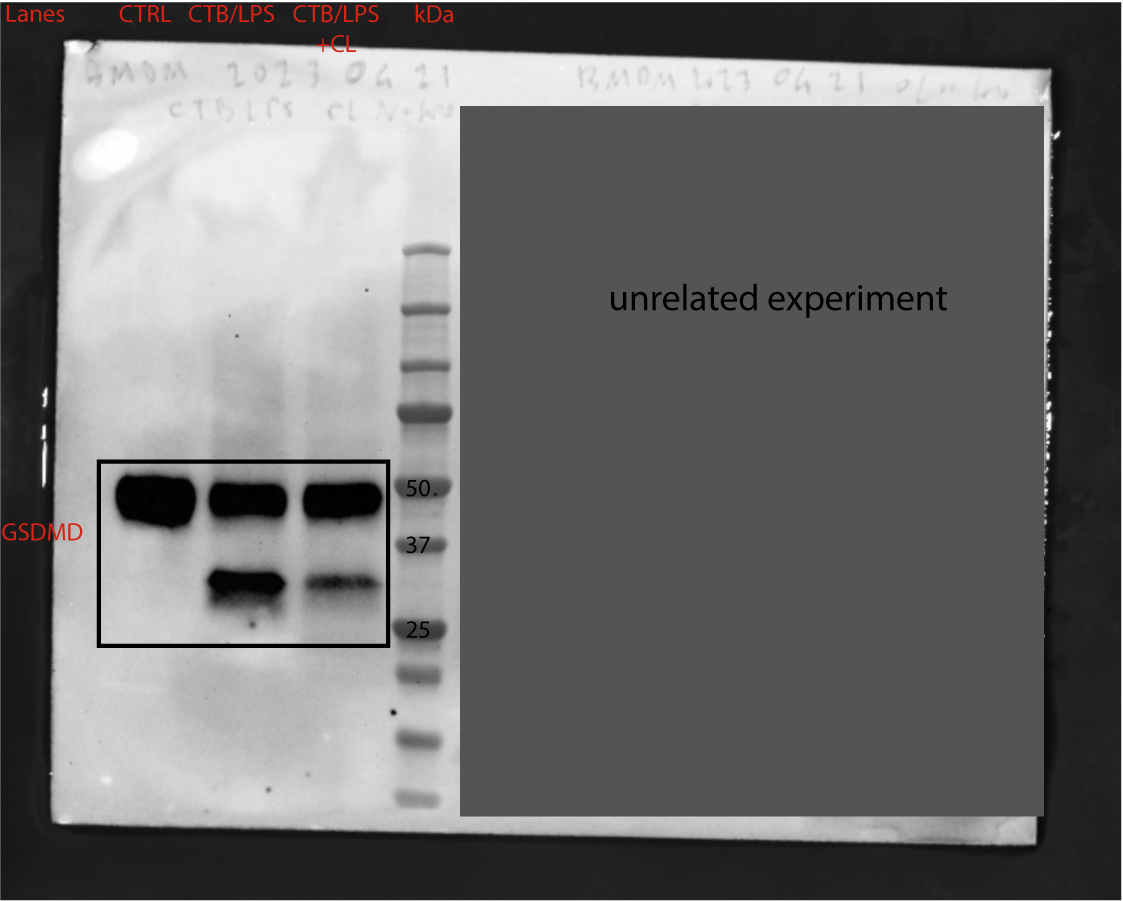

Supplement: Supplementary file 3 — Source data Fig. 1 [file 44318_2025_507_MOESM3_ESM.zip › Figure 1/1C/GSDMD-HRP 311sec.tif]

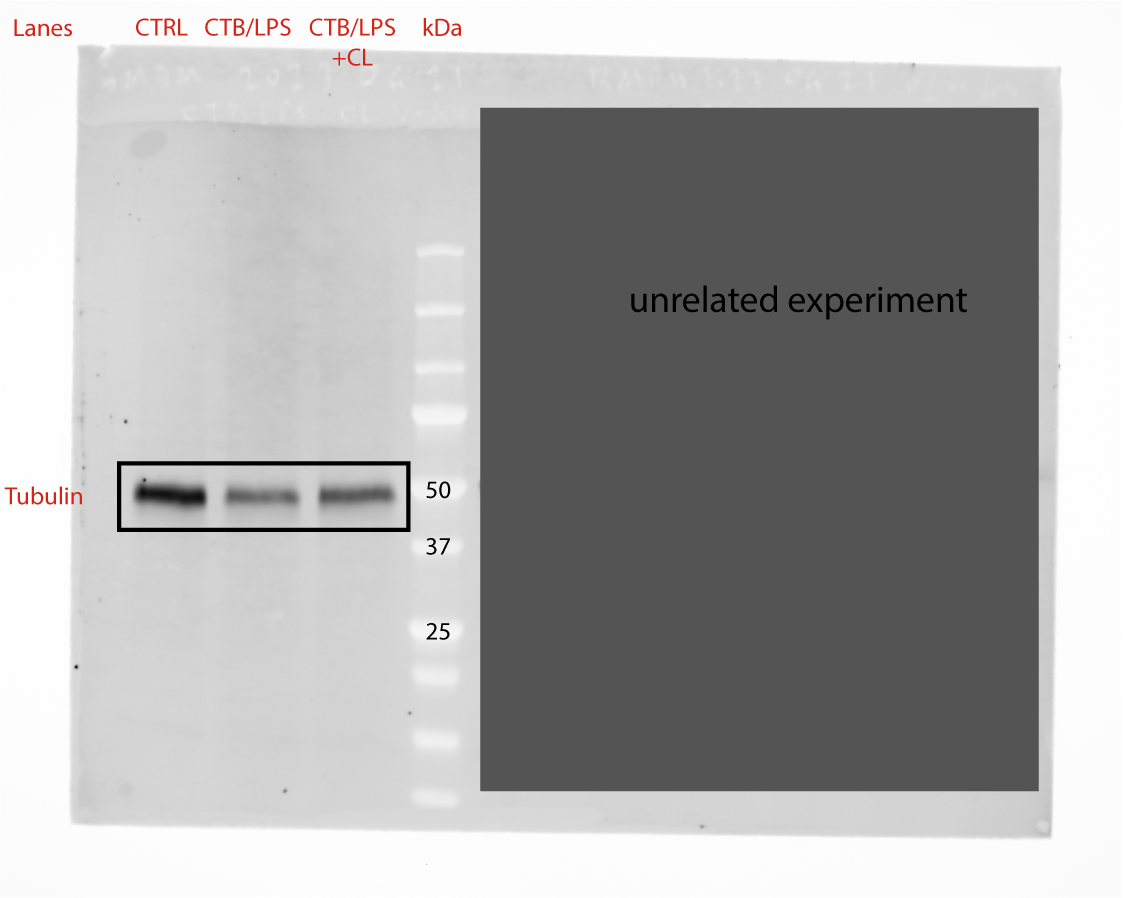

Supplement: Supplementary file 3 — Source data Fig. 1 [file 44318_2025_507_MOESM3_ESM.zip › Figure 1/1C/Tubulin Rhodamine 46sec.tif]

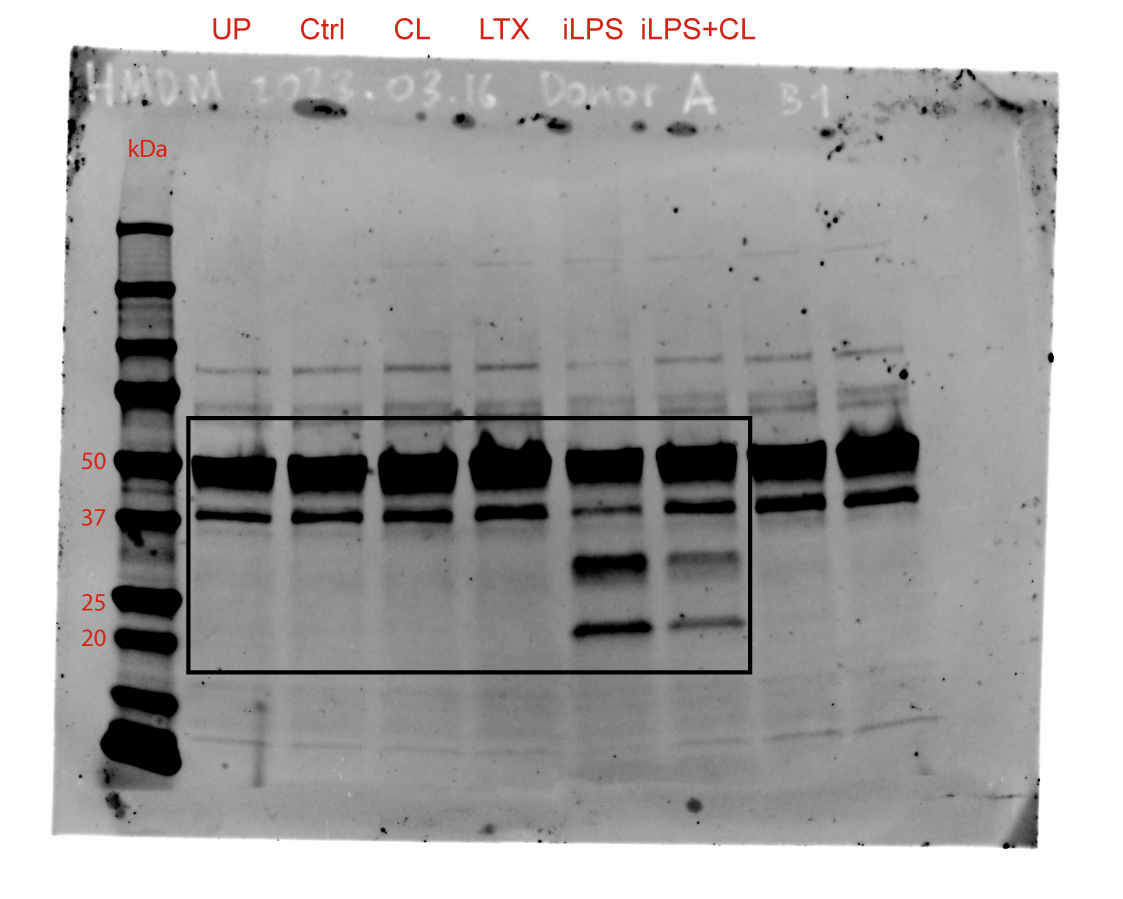

Supplement: Supplementary file 4 — Source data Fig. 2 [file 44318_2025_507_MOESM4_ESM.zip › Figure 2/2A/GSDMD Rb StarBright B700 239sec.tif]

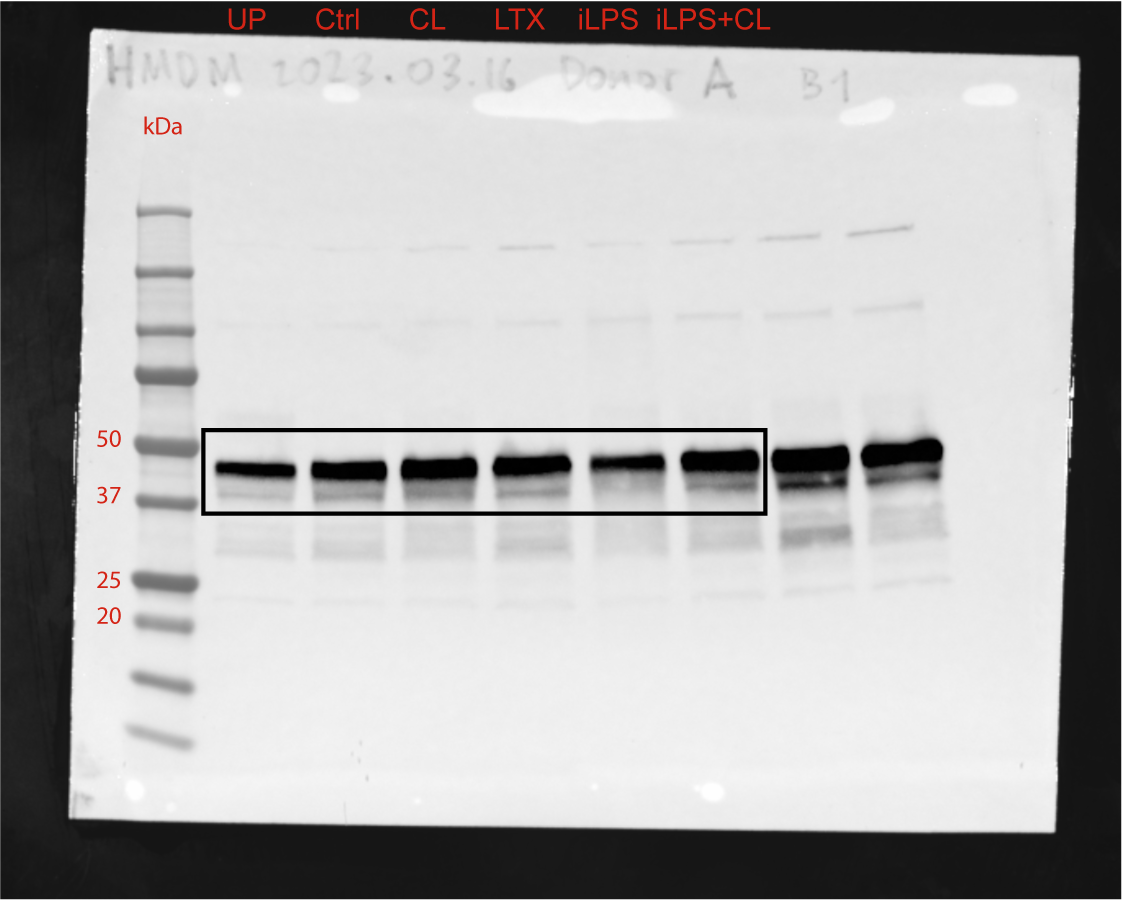

Supplement: Supplementary file 4 — Source data Fig. 2 [file 44318_2025_507_MOESM4_ESM.zip › Figure 2/2A/CASP4 Ms HRP 10sec.tif]

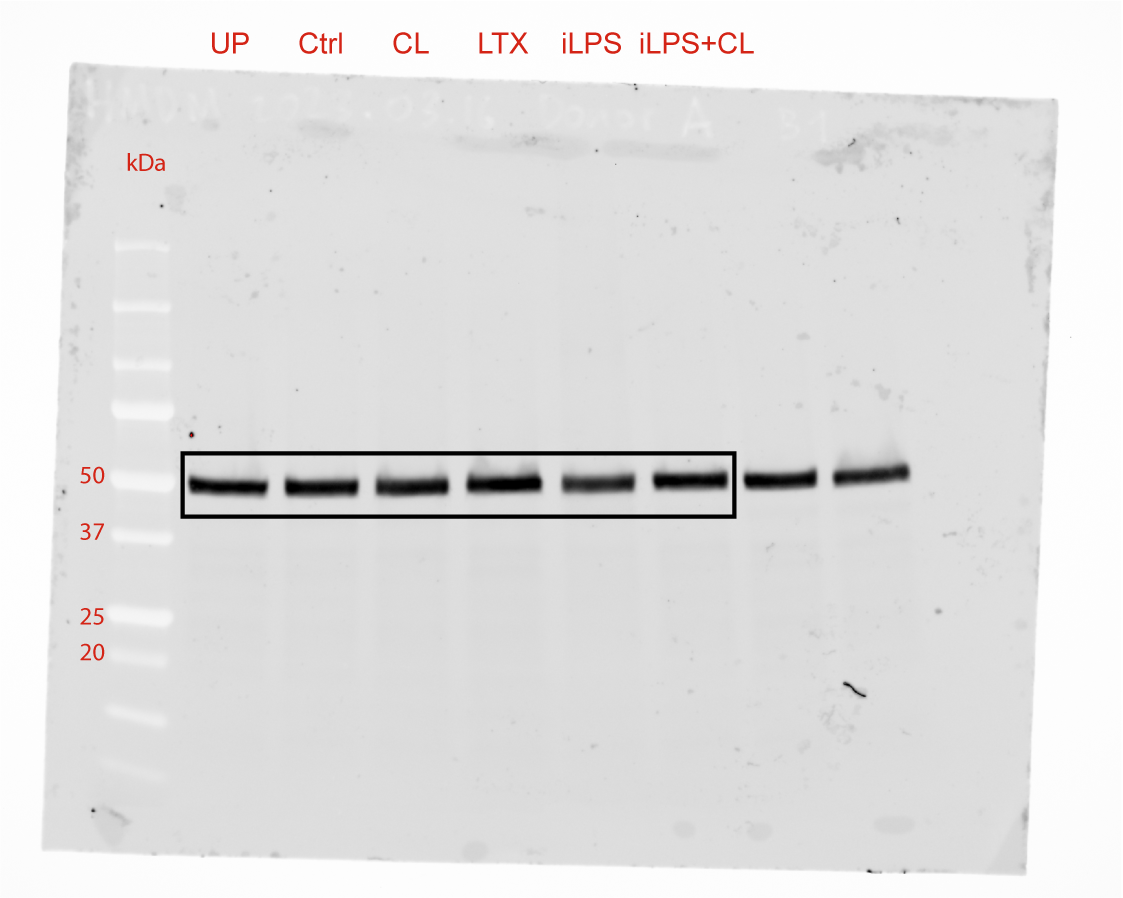

Supplement: Supplementary file 4 — Source data Fig. 2 [file 44318_2025_507_MOESM4_ESM.zip › Figure 2/2A/Tubulin Rho 44sec.tif]

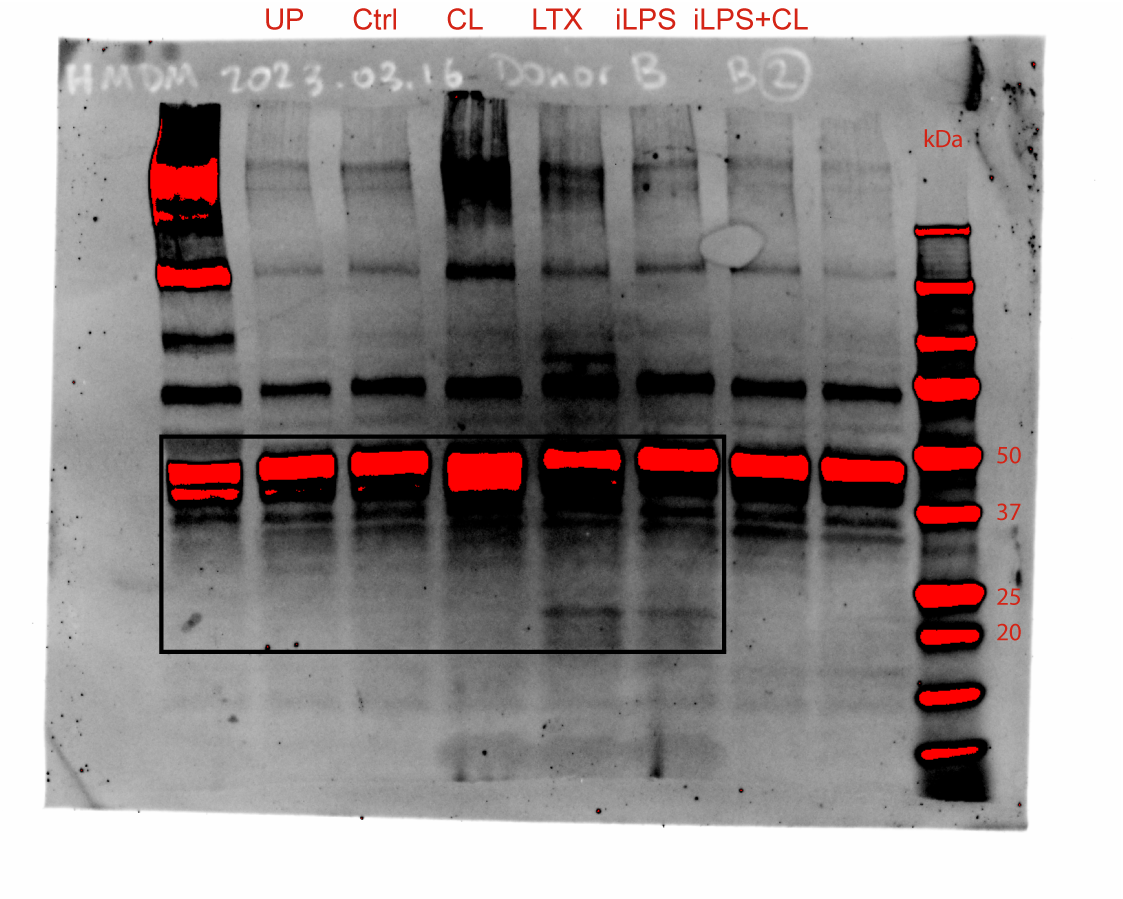

Supplement: Supplementary file 4 — Source data Fig. 2 [file 44318_2025_507_MOESM4_ESM.zip › Figure 2/2A/Casp1 StarBright B700 247sec.tif]

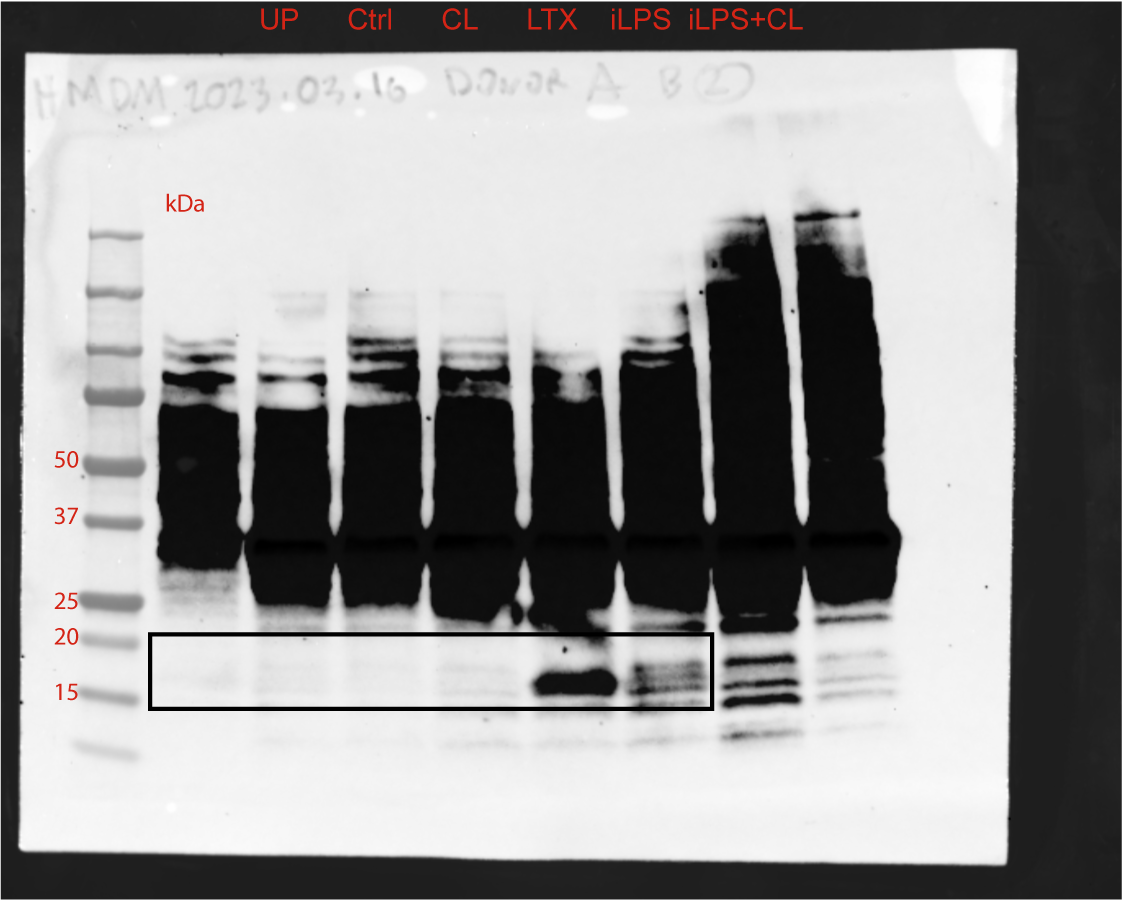

Supplement: Supplementary file 4 — Source data Fig. 2 [file 44318_2025_507_MOESM4_ESM.zip › Figure 2/2A/IL1b Goat HRP 600sec.tif]

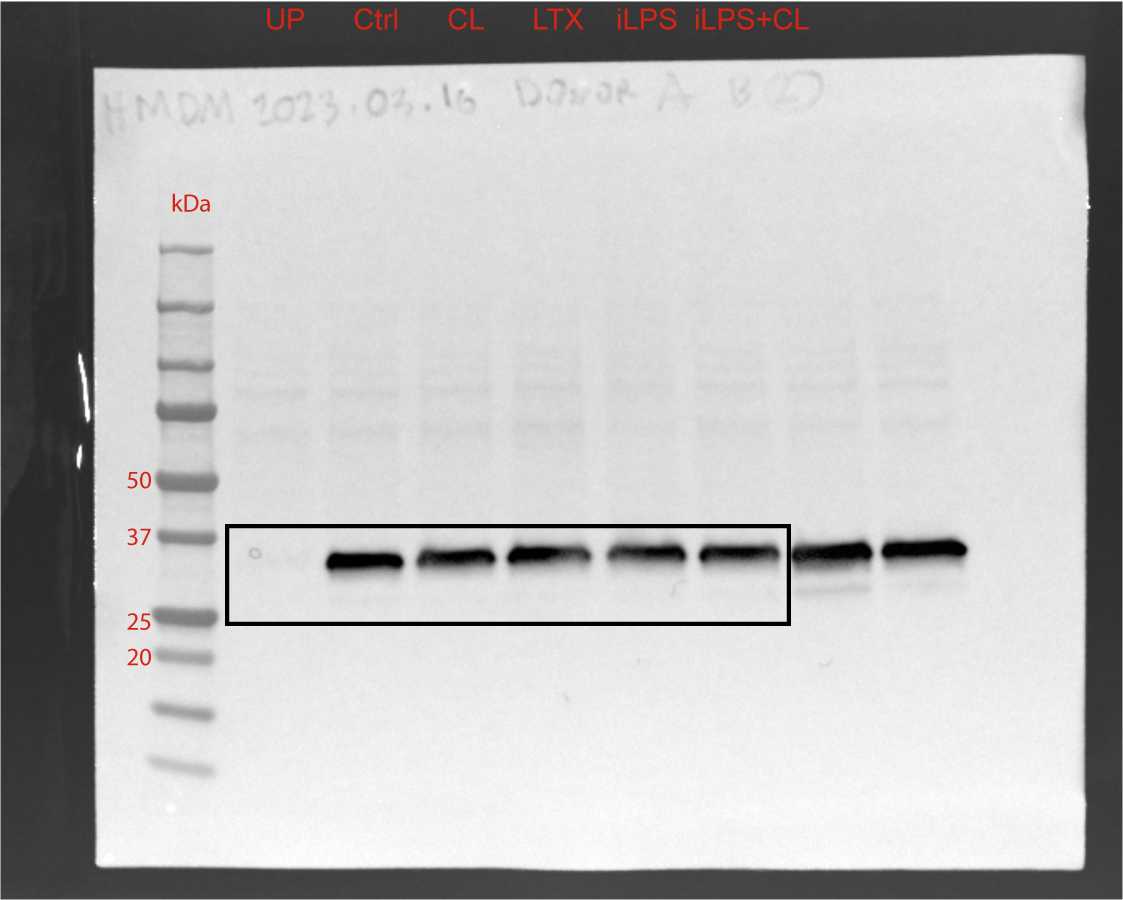

Supplement: Supplementary file 4 — Source data Fig. 2 [file 44318_2025_507_MOESM4_ESM.zip › Figure 2/2A/IL1b Goat HRP 15sec.tif]

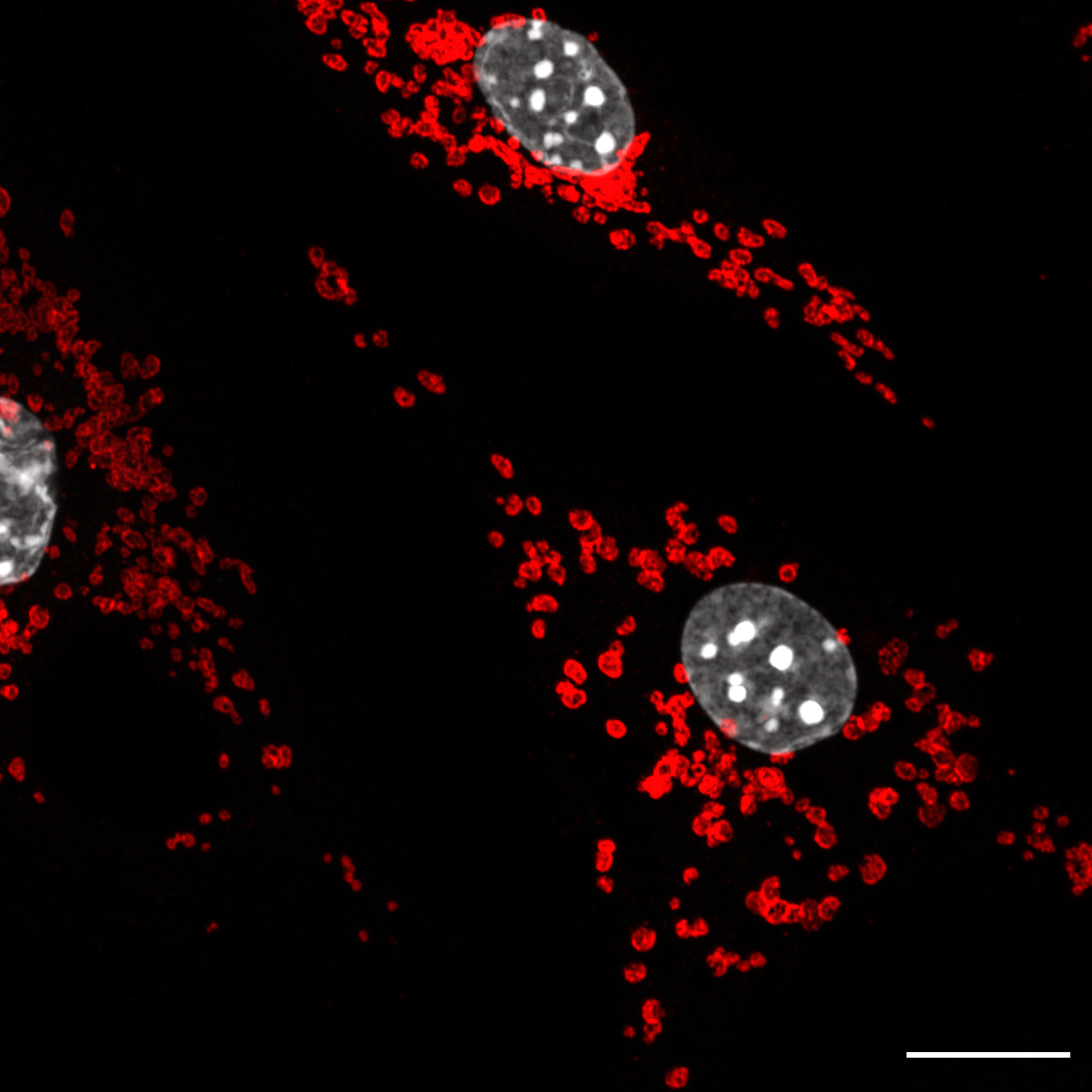

Supplement: Supplementary file 4 — Source data Fig. 2 [file 44318_2025_507_MOESM4_ESM.zip › Figure 2/2C/Control_flatten-MAX_X520-BMM-CT-488-Tom20_594-DAPI-2_Airyscan Processing.tif (RGB).tif]

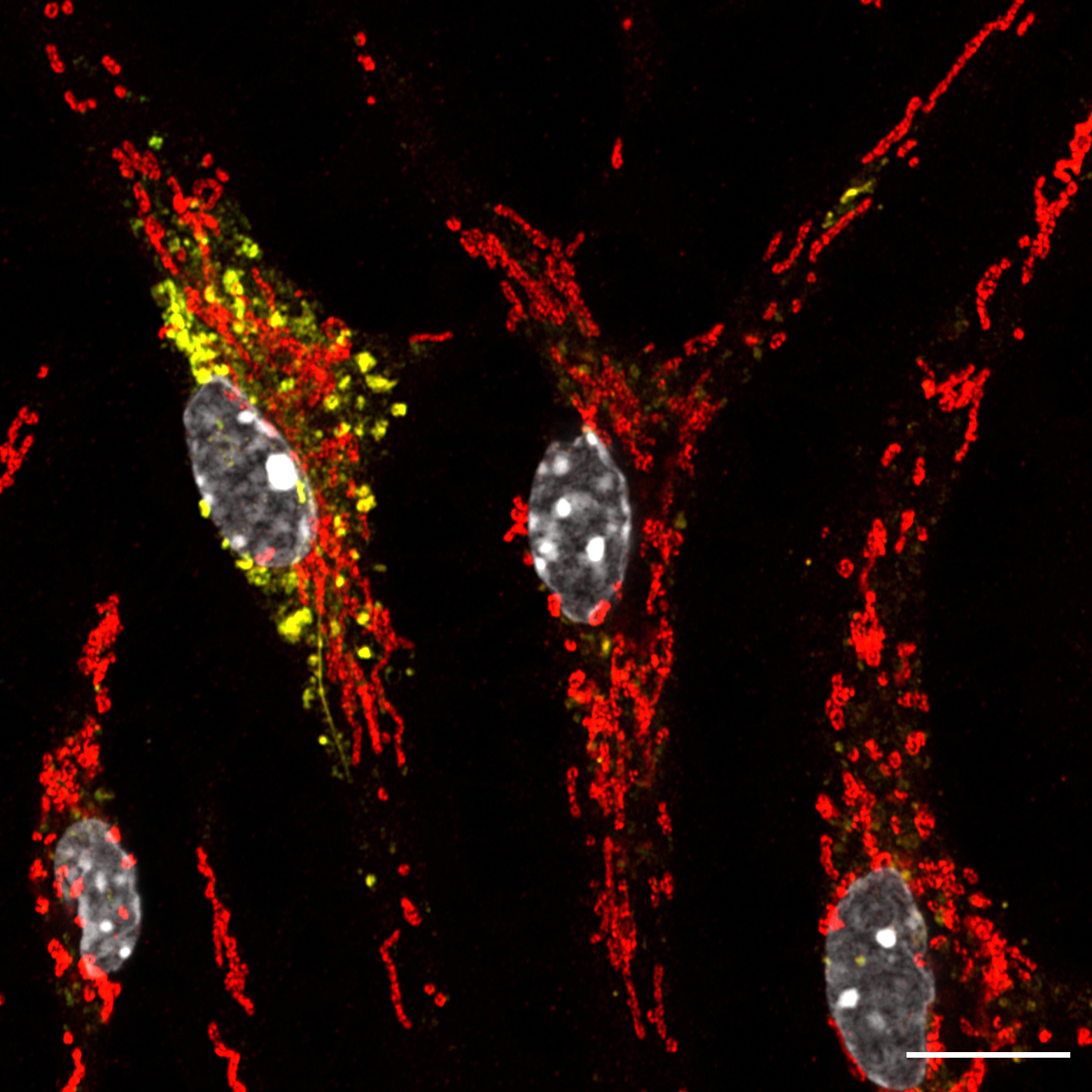

Supplement: Supplementary file 4 — Source data Fig. 2 [file 44318_2025_507_MOESM4_ESM.zip › Figure 2/2C/CL-Bodipyflatten-MAX_X520-BMM-CL-488-Tom20_594-DAPI-1_Airyscan Processing-1.tif (RGB).tif]

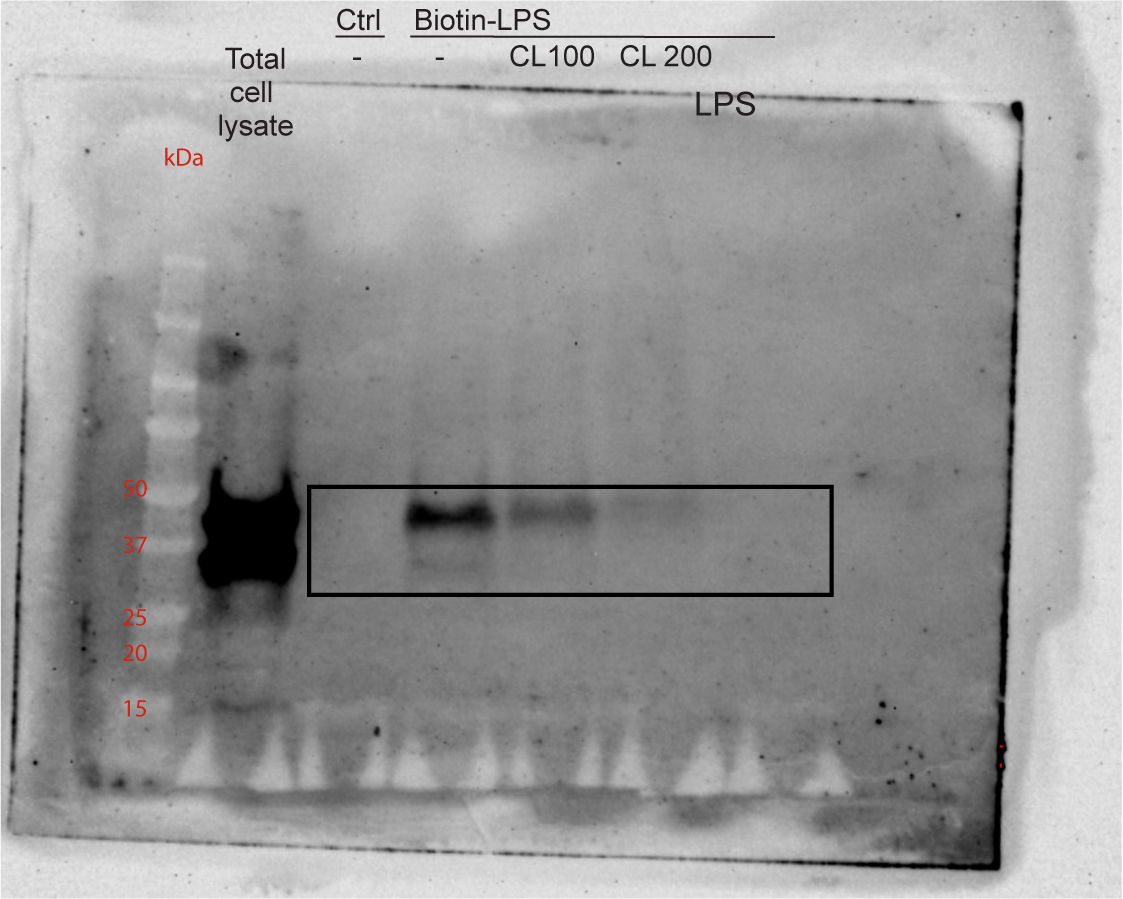

Supplement: Supplementary file 4 — Source data Fig. 2 [file 44318_2025_507_MOESM4_ESM.zip › Figure 2/2D/Bound CASP11 Rat HRP.tif]

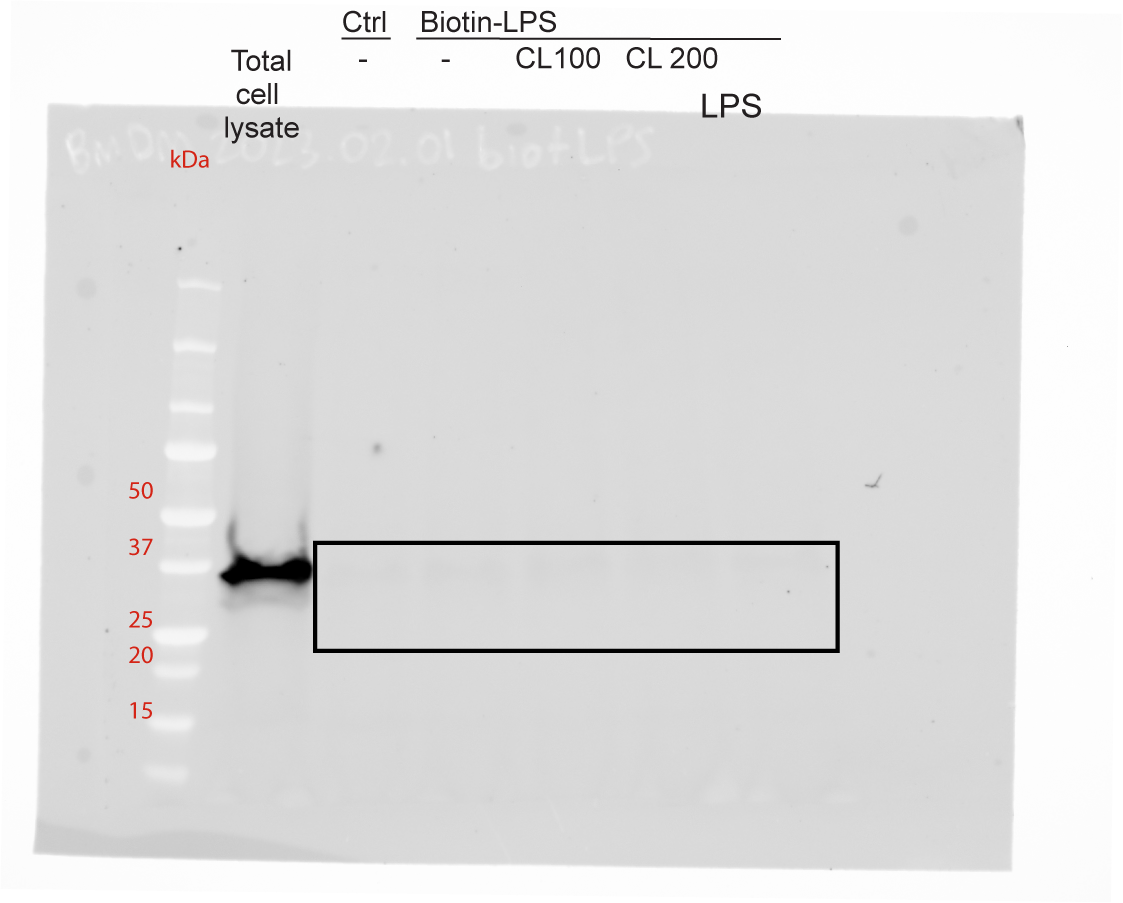

Supplement: Supplementary file 4 — Source data Fig. 2 [file 44318_2025_507_MOESM4_ESM.zip › Figure 2/2D/Bound GAPDH.tif]

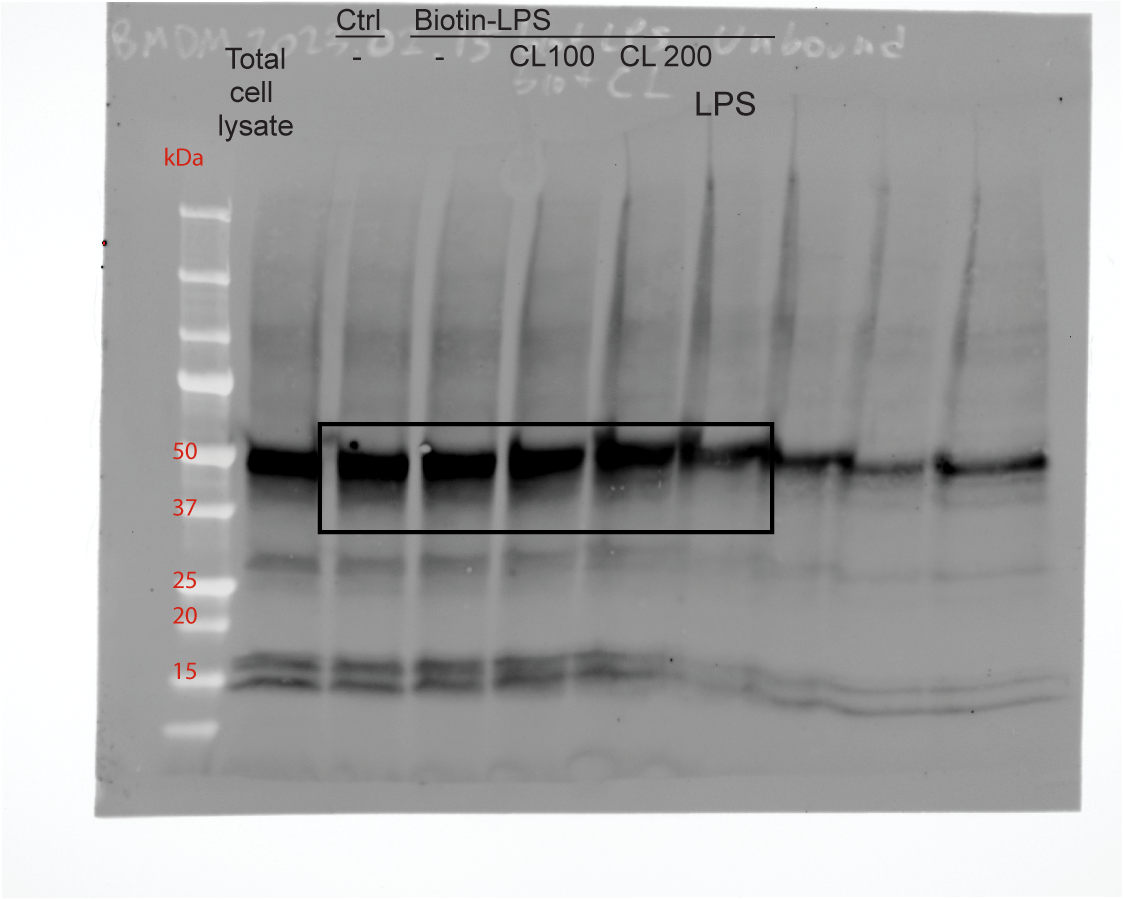

Supplement: Supplementary file 4 — Source data Fig. 2 [file 44318_2025_507_MOESM4_ESM.zip › Figure 2/2D/Unbound CASP11.tif]

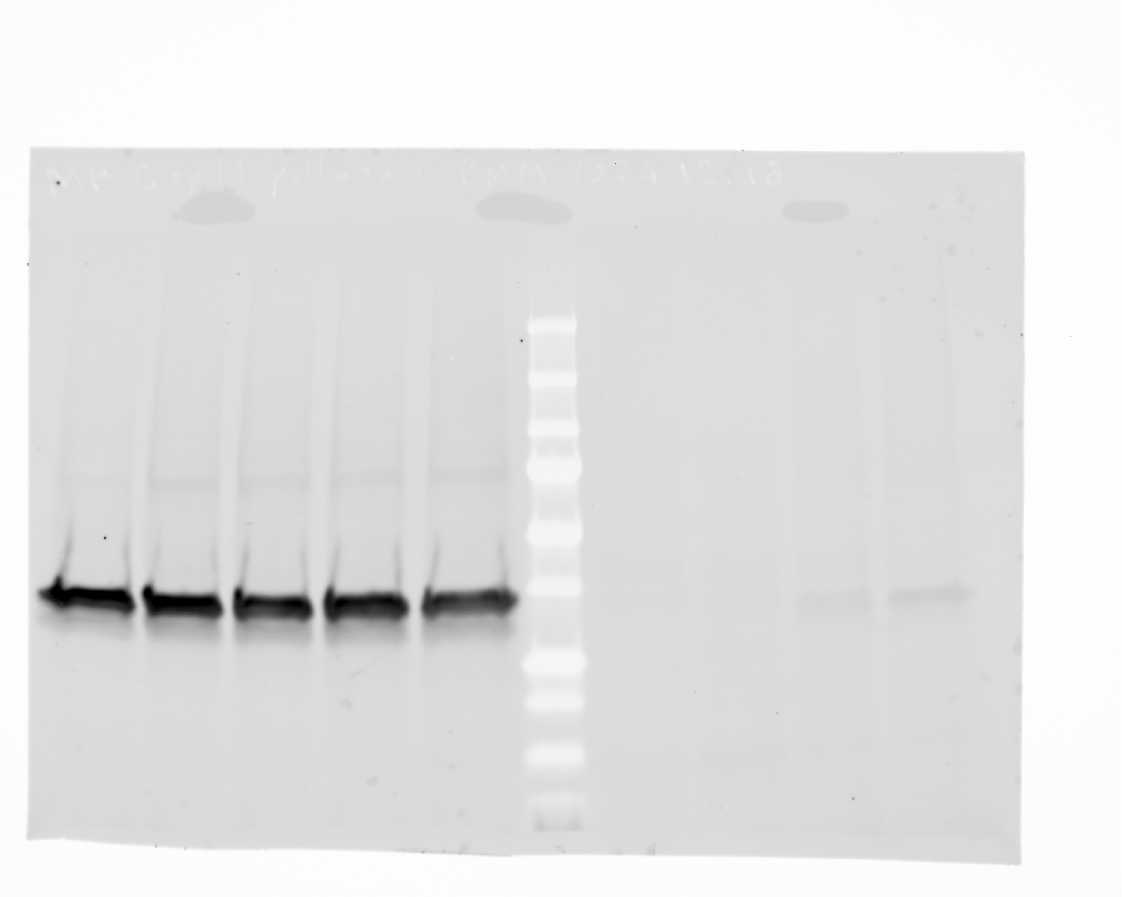

Supplement: Supplementary file 4 — Source data Fig. 2 [file 44318_2025_507_MOESM4_ESM.zip › Figure 2/2D/pull down BMDM 2024 12 18 GAPDH Rhodamine 2024-12-21 42.311s(Rhodamine).tif]

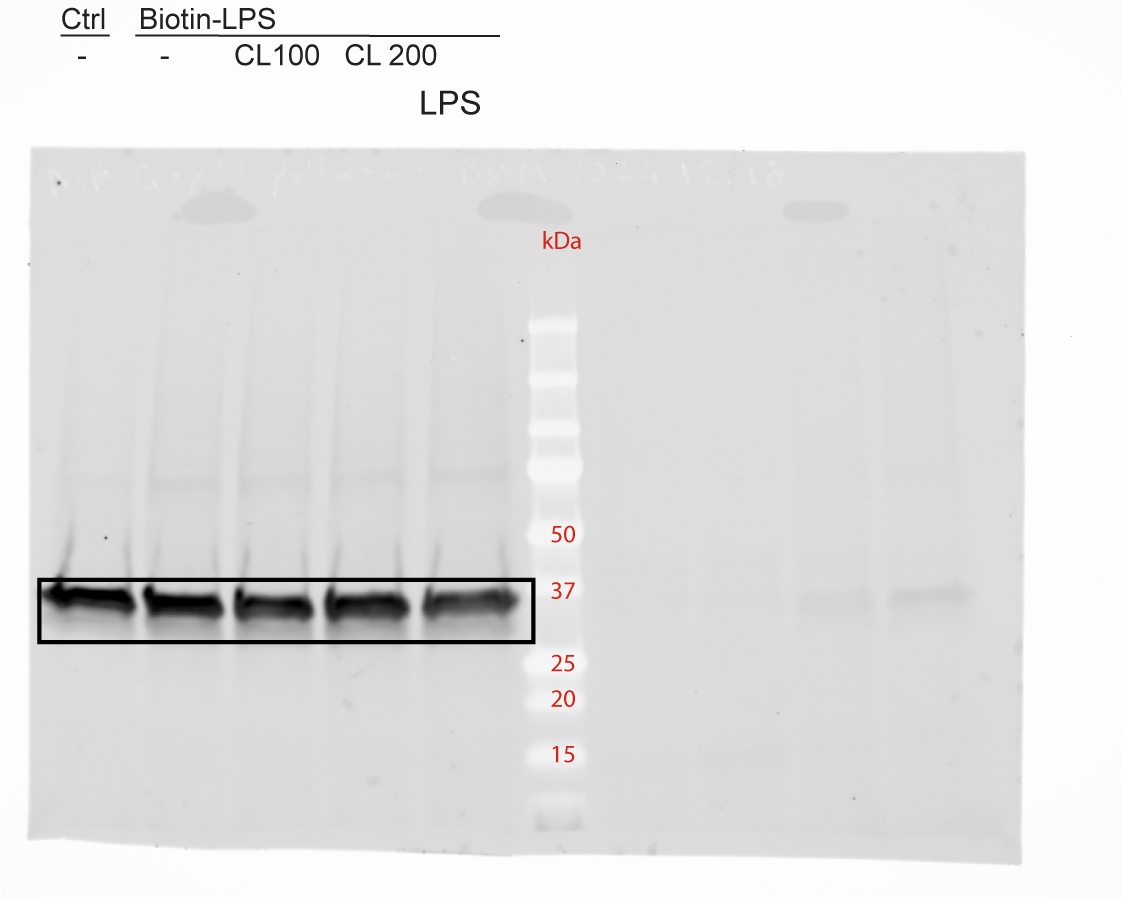

Supplement: Supplementary file 4 — Source data Fig. 2 [file 44318_2025_507_MOESM4_ESM.zip › Figure 2/2D/Unbound GAPDH.tif]

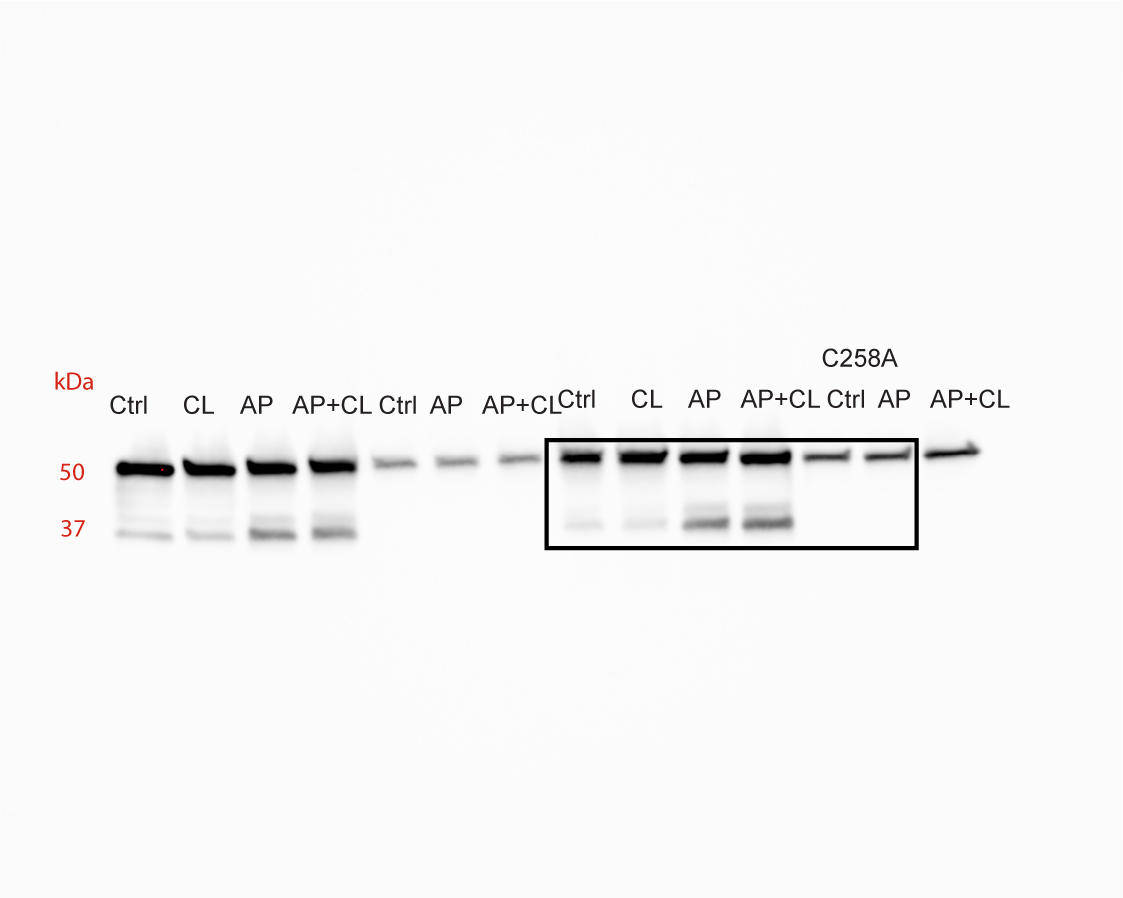

Supplement: Supplementary file 4 — Source data Fig. 2 [file 44318_2025_507_MOESM4_ESM.zip › Figure 2/2F/DeltaCARD V5.tif]

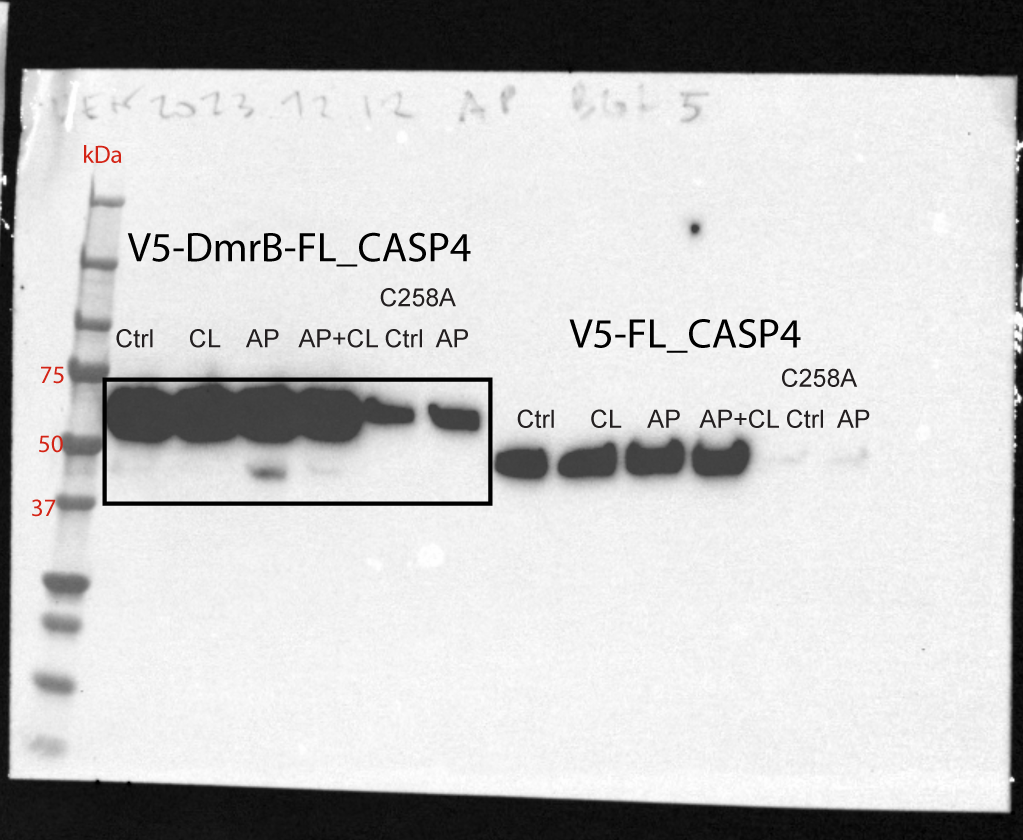

Supplement: Supplementary file 4 — Source data Fig. 2 [file 44318_2025_507_MOESM4_ESM.zip › Figure 2/2E/FL V5.tif]
