# Supplementary material for: Cardiolipin inhibits the non-canonical inflammasome by preventing LPS binding to caspase-4/11
Source: EMBO J. 2025 Jul 16;44(16):4419–42. doi: 10.1038/s44318-025-00507-z (PMC12361528; doi:10.1038/s44318-025-00507-z)
Supplement: Supplementary file 7 — Expanded View Figures [file 44318_2025_507_MOESM7_ESM.pdf]

Expanded View Figures

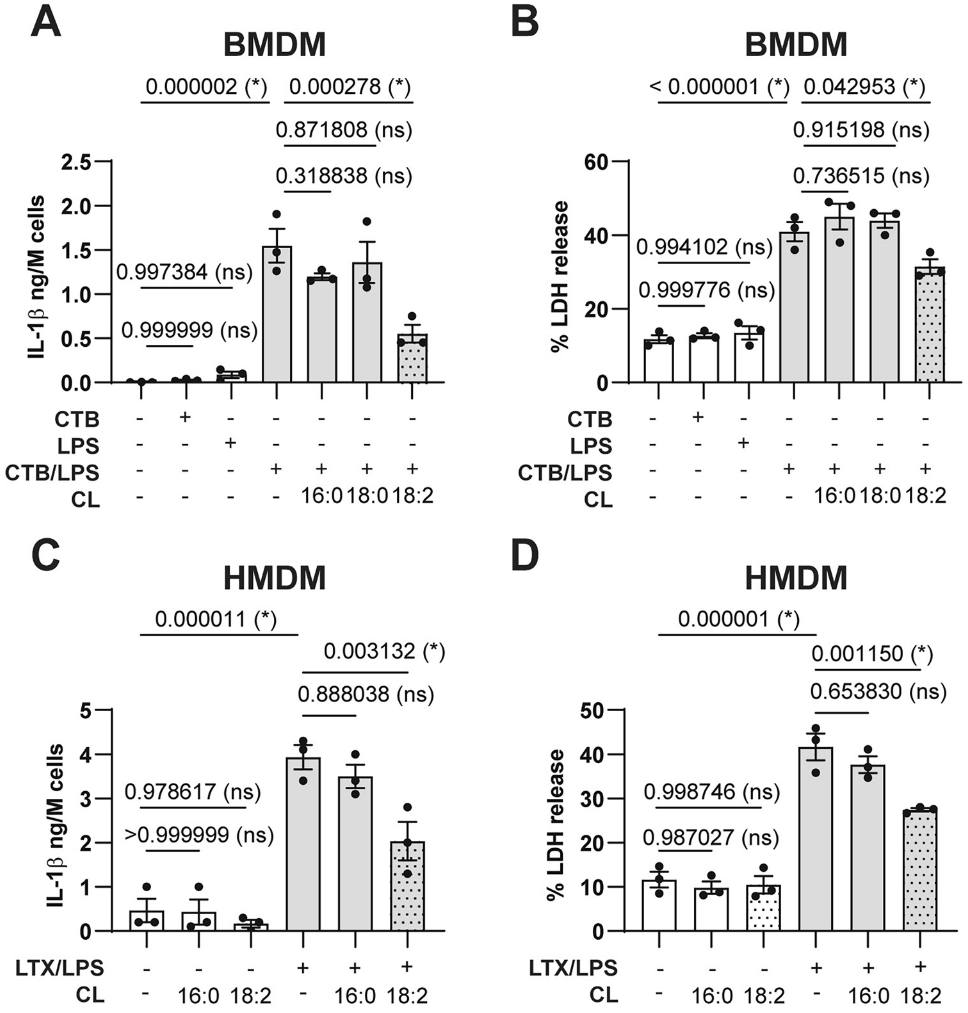

Figure EV1. Unsaturated 18:2 but not saturated 16:0 and 18:0 CL inhibits noncanonical inflammasome signalling.

(**A**, **B**) WT BMDM were incubated for 4 h with 1 µg/mL Pam_3CSK_4. Cell culture medium was then replaced with OptiMEM plus 20 µg/mL CTB, 2 µg/mL LPS, or 20 µg/mL CTB complexed with 2 µg/mL LPS (CTB/LPS), in the absence or presence of 10 µM saturated 16:0 CL, saturated 18:0 CL or unsaturated 18:2 CL. Cells were incubated for 18 h. Cleaved IL-1β was quantified in cell supernatants by ELISA (**A**). LDH release was quantified by cytotoxicity assay (**B**). (**C**, **D**) HMDM were incubated for 4 h with 0.1 µg/mL LPS. Cell culture medium was then replaced with OptiMEM or 20 µg/mL LTX complexed with 2 µg/mL LPS (LTX/LPS) in OptiMEM, in the absence or presence of 10 µM saturated 16:0 CL or unsaturated 18:2 CL. Cells were incubated for 4 h. Cleaved IL-1β was quantified in cell supernatants by ELISA (**C**). LDH release was quantified by cytotoxicity assay (**D**). Data information: Each symbol is the mean of three technical replicates from an independent biological replicate. Bars are the mean of three independent biological replicates ($n = 3$) ± SEM. Statistical analysis: Data were verified for normality using a Shapiro–Wilk test and analysed by one-way ANOVA Šídák's multiple comparisons test. *P* values are reported above bars. Statistical significance was defined as follows: significant difference for $P < 0.05$ (*), not significant for $P \geq 0.05$ (ns).

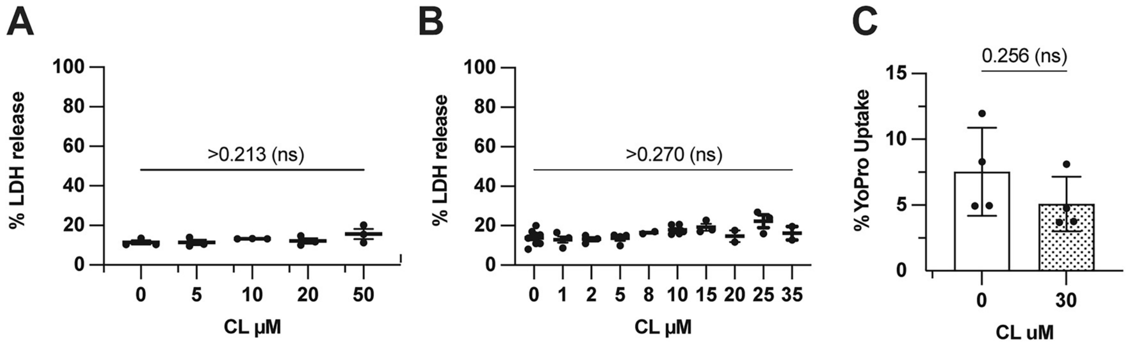

Figure EV2. CL does not induce cell death.

HMDM from healthy donors (**A**) or BMDM from wild-type mice (**B**, **C**) were incubated for 4 h with 1 µg/mL Pam_3CSK_4. Cell culture medium was then replaced with OptiMEM (0) or increasing CL concentrations (1 to 50 µM), and cells were incubated for 18 h. Lytic cell death was evaluated by LDH activity in supernatants (**A**, **B**) or YoPro uptake by lytic cells (**C**) and reported here as the percentage of cell lysis induced by 0.1% Triton. Data information: Each symbol is the mean of three technical replicates from an independent biological replicate. Bars are the mean of two or more independent biological replicates ($n = 2$ to 7) ± SEM. Statistical analysis: Data were verified for normality using a Shapiro–Wilk test and analysed by (**A**) one-way ANOVA, (**B**) Kruskal–Wallis test compared to control (0) ($n = 2$ not included in the statistical analysis), (**C**) unpaired *t* test. *P* values are reported above bars. Statistical significance was defined as follows: significant difference for $P < 0.05$ (*), not significant for $P \geq 0.05$ (ns).

 © The Author(s)

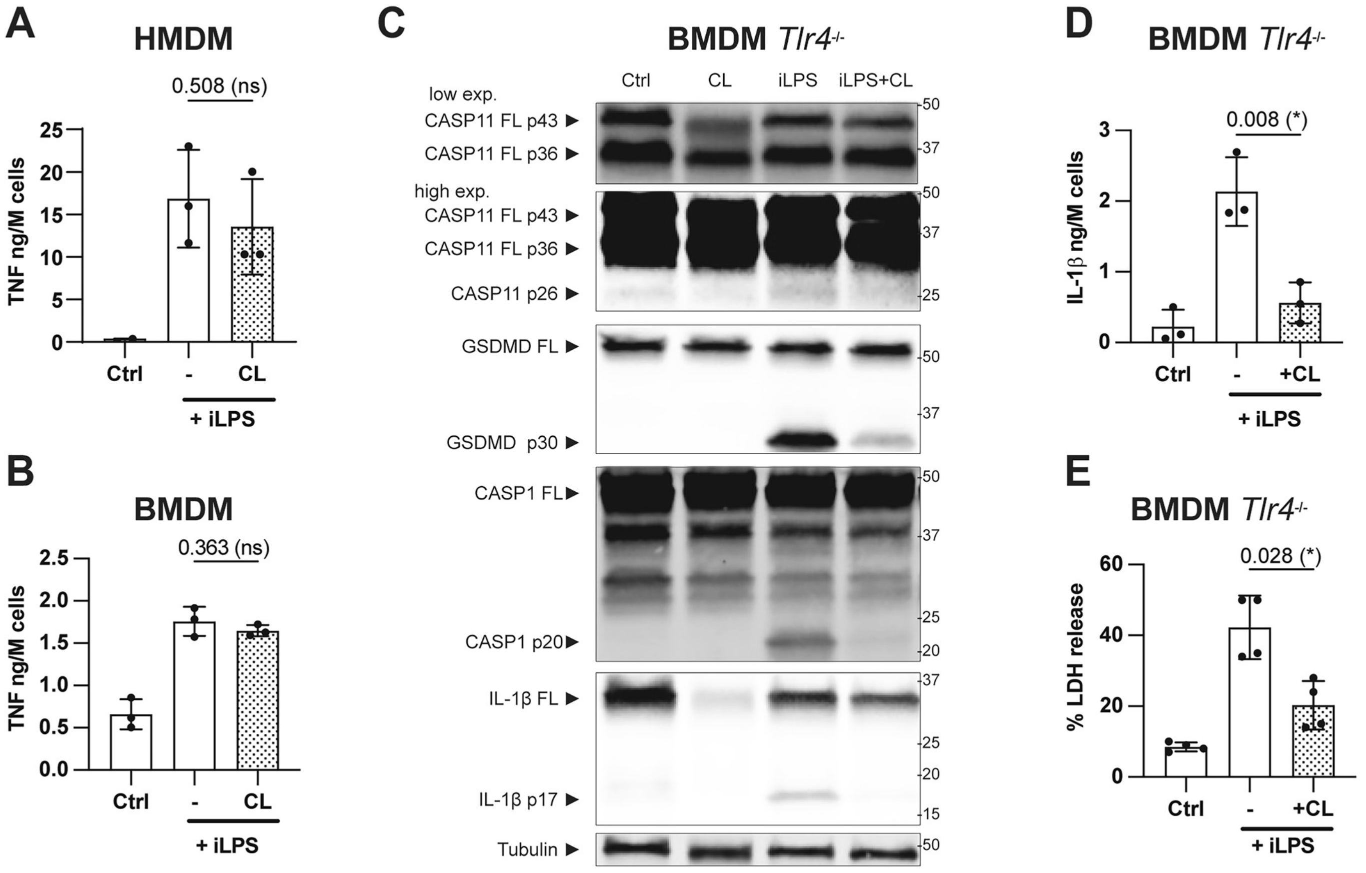

Figure EV3. CL inhibits CASP11 activation independently of TLR4.

HMDM from healthy donors (**A**), and BMDM from wild-type (**B**) or *Tlr4*$^{-/-}$ (**C–E**) mice were incubated for 4 h with 1 µg/mL Pam_3CSK_4. Cell culture medium was then replaced with OptiMEM or the noncanonical inflammasome activator LPS complexed with 0.25 % v/v LTX (**A**) or 20 µg/mL CTB (**B–E**) (iLPS) in the presence of HEPES (−) or 10 µM CL. Released TNF and cleaved IL-1β were measured in supernatants by ELISA (**A**, **B**, **D**). CASP11, GSDMD, IL-1β cleavage, and tubulin expression were assessed by western blot (**C**). LDH release was quantified by cytotoxicity assay (**E**). Data information: The blot is representative of three independent biological replicates ($n = 3$). Each symbol is the mean of three technical replicates from an independent biological replicate. Bars are the mean of three or more independent biological replicates ($n = 3$ to 4) ± SEM. Statistical analysis: Data were verified for normality using a Shapiro–Wilk test and analysed by (**A**, **E**) Mann–Whitney test, (**B**, **D**) unpaired *t* test. *P* values are reported above bars. Statistical significance was defined as follows: significant difference for $P < 0.05$ (*), not significant for $P \geq 0.05$ (ns).

© The Author(s)

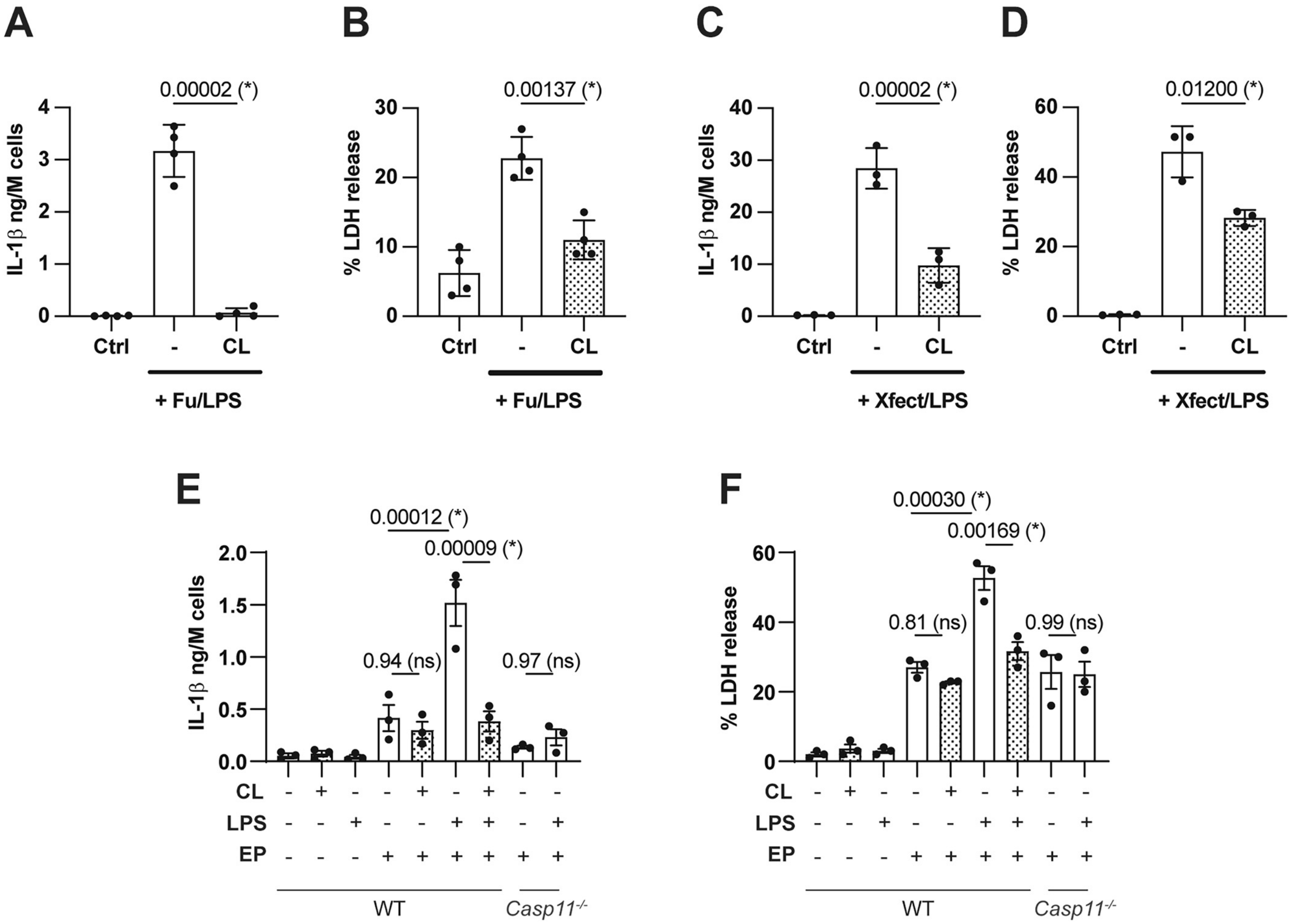

Figure EV4. CL inhibits the noncanonical inflammasome regardless of the delivery system used for LPS.

(**A–D**) BMDM from WT mice were incubated for 4 h with 1 µg/mL Pam_3CSK_4. Cell culture medium was then replaced with OptiMEM, or 2 µg/mL of the noncanonical inflammasome activator LPS from *Escherichia coli* B4 strain (EC-B4) complexed with 0.5% FuGENE HD (**A**, **B**) or 1.2% Xfect (**C**, **D**) (iLPS) in the presence of HEPES (−) or 10 µM CL. Cells were incubated for 18 h (**A**, **B**) or 4 h (**C**, **D**). Cleaved IL-1β was quantified in cell supernatants by ELISA. LDH release was quantified by cytotoxicity assay. (**E**, **F**) BMDM from wild-type or *Casp11*$^{-/-}$ mice were incubated for 4 h with 1 µg/mL Pam_3CSK_4. Cell culture medium was then replaced with OptiMEM in the presence of HEPES (−), 10 µM CL, or 2 µg/mL of LPS. Cells were left untouched or electroporated (+ EP), then fresh OptiMEM was added, and cells were incubated for 4 h. Cleaved IL-1β was quantified in cell supernatants by ELISA. LDH release was quantified by cytotoxicity assay. Data information: Each symbol is the mean of technical triplicates from an independent biological replicate. Bars are the mean of three or more independent biological replicates ($n = 3$ to 4) ± SEM. Statistical analysis: Data were verified for normality using a Shapiro–Wilk test and analysed by (**A–D**) unpaired *t* test, (**E**, **F**) one-way ANOVA Šídák's multiple comparisons test. *P* values are reported above bars. Statistical significance was defined as follows: significant difference for $P < 0.05$ (*), not significant for $P \geq 0.05$ (ns).

 © The Author(s)

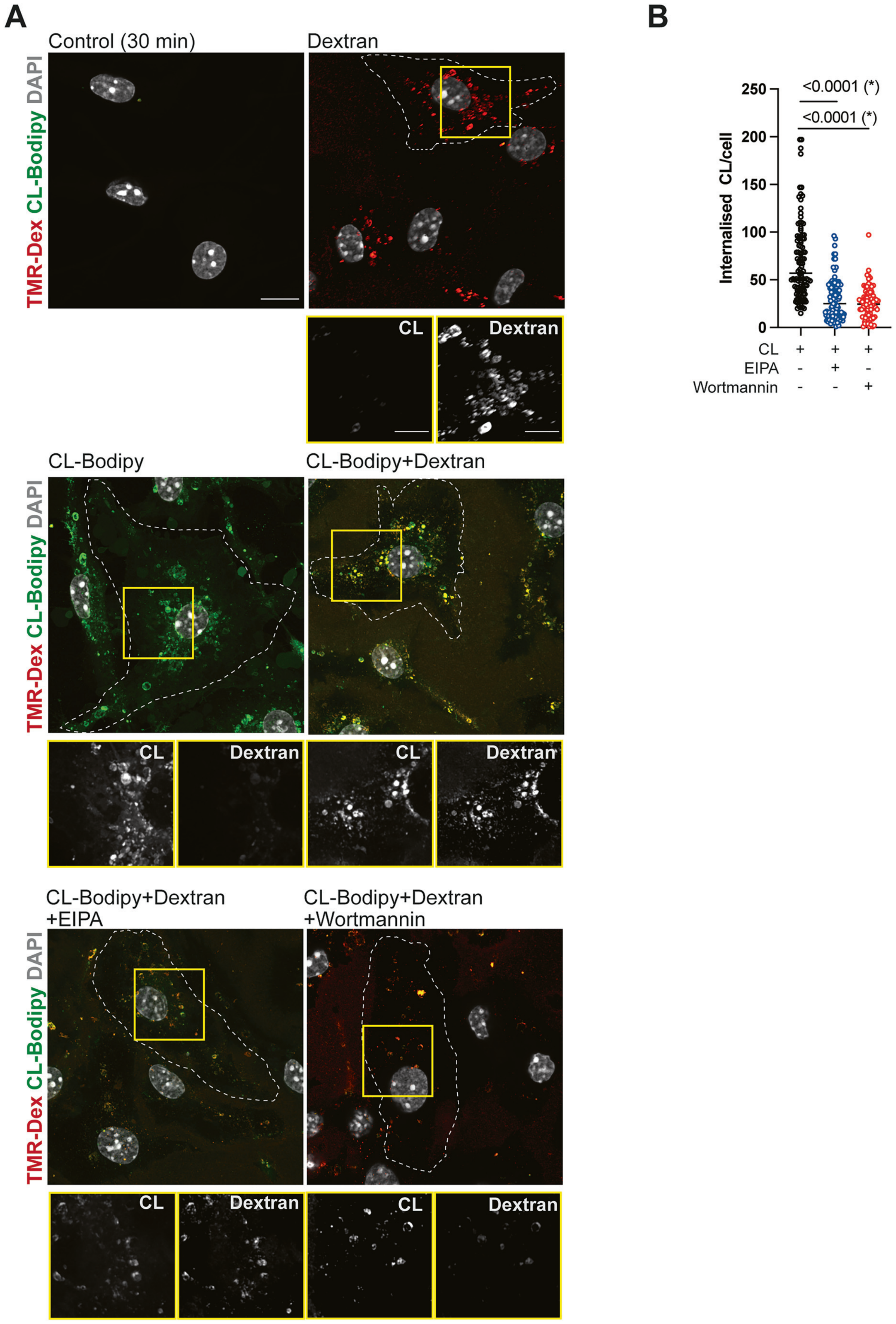
A
Control (30 min)
Dextran
TMR-Dex CL-Bodipy DAPI
CL
Dextran
CL-Bodipy
CL-Bodipy+Dextran
CL
Dextran
CL
Dextran
CL-Bodipy+Dextran
+EIPA
CL-Bodipy+Dextran
+Wortmannin
CL
Dextran
CL
Dextran
B
Internalised CL/cell
250
200
150
100
50
0
<0.0001 (*)
<0.0001 (*)
CL + + +
EIPA - + -
Wortmannin - - +

Figure EV5. CL accesses the cell interior by endocytosis to inhibit CASP11 activation in BMDM.

(A) BMDM were primed for 4 h with 1 µg/mL Pam_3CSK_4 and incubated for 1 h in OptiMEM with EIPA (10 µM) or Wortmannin (10 µM) prior to the addition of HEPES (Control), 10 µM of CL liposomes containing 1% (w/w) TopFluor CL (CL-BODIPY), or 100 µg/mL 70 kDa TMR-dextran for 30 min. Macrophages were immunostained with phalloidin (for quantification) and DAPI (grey). Images are fixed-Airyscan confocal imaging of DAPI (grey), TMR-dextran (red) and CL-BODIPY (green). Cell outlines were drawn from brightfield. Data information: Images are maximum intensity projections of Z-stacks acquisitions and representative of three independent experiments ($n = 3$). Scale bar = 10 µm and 5 µm in inset panels. (**B**) The graph is the quantification of CL uptake: for quantification, images were segmented based on phalloidin staining, and internalised TopFluor CL was quantified in Fiji. Each dot represents the number of BODIPY-positive vesicles per cell from three independent biological replicates ($n = 3$). Statistical analysis: Data were verified for normality using a Shapiro–Wilk test and analysed by Mann–Whitney test. *P* values are reported above bars. Statistical significance was defined as follows: significant difference for $P < 0.05$ (*), not significant for $P \geq 0.05$ (ns).

© The Author(s)
